# Round the Hospitals

**Published:** 1918-11-16

**Authors:** 


### ROUND THE HOSPITALS.

THE sense of glory and the amazing poignancy of the last few days undoubtedly reached highest pitch in the hospitals. We heard of one where, when the news came (rather prematurely) of the signing of the armistice, the whole establishment broke into an orgy of song and dance, the matron herself beating time, while the wounded scrambled out of bed, sitting or lying ecstatically drumming on whatever came to hand to swell the chorus. With many variants through diverse moods of expression, these scenes, we may be sure, were enacted in every hospital in this country, and in many another country beside. The magnificent spirit of cheery endurance and victory over suffering which the hospitals have radiated through the dark years has changed to a very beautiful spirit of entire content. To these wounded men, and to those who have upheld them through all, the heart of the nation turns first in the moment of high emotion.

---

MANY nursing sisters were to be observed in the monstrous crowds which visited Buckingham Palace on Monday by a common instinct, and then turned to offer their thanksgiving in the Abbey. None of that crowd will ever forget the sounding note of rejoicing which burst forth again and again as choir and organ and worshippers realised some vivid sentence in the accustomed office which had suddenly assumed a new and tremendous meaning. One such moment came in the response to "Give peace in our time, O Lord," when the voices rang out slowly and in unison with a thundering accompaniment, "Because there is none other that

**ROUND THE HOSPITALS—(*continued*).**

fighteth for us, but only Thou, O God." A few yards away members of the House of Commons were united in a spontaneous tribute of prayer and praise. If ever in the course of our history men's minds have been lifted to supreme realisation of a destiny controlled by God, it was there and then.

At the Investiture held at Buckingham Palace on November 9 the King personally conferred the decoration of the Royal Red Cross, First Class, on Sister Elizabeth Rogers, Q.A.I.M.N.S.(R.); Assistant Matron Florence Carter and Sister Mildred Oakley, T.F.N.S.; Matron Alice Reeves, C.N.S. His Majesty conferred the Royal Red Cross, Second Class, on Assistant Matron Mabel Woodfin and Sister Charlotte Robertson, B.R.C.S.; Nursing Sister Florence Perdue, Doughty White Unit; Mrs. Frances Roberts, Miss Edyth Taylor, and Mrs. Bella Taylor, V.A.D.

An amusing exhibition of the true inwardness of the vitality of the Central Committee for the State Registration of Nurses is illustrated this week by a correspondence which has been sent to us between the College of Nursing and this Committee. The Council of the College, being informed on the authority of Major Chapple that the Central Committee had received copies of the seventh draft of the Bill for the State Registration of Nurses, wrote to the latter Committee stating that if the members felt that any difficulty was caused by Clause 5 (3) of the seventh draft of the Bill the College would be willing to delete that clause. A long reply dated November 1 was received by the College, stating that the Committee is unable to support the Bill proposed by the College even if Clause 5 (3) is deleted. The letter then repeats at length old points, the substance of which has been demolished many times in the course of the various discussions which have taken place between the two bodies, and adds a polite intimation in effect that the College as it stands is anathema to the members of the Central Committee, though that Committee recognises the educational importance of the College.

Some light is thrown on this matter in recent numbers of Mrs. Fenwick's paper, where the report of the meeting of the Central Committee is published in the issue of November 2, at which five resolutions drafted and adopted by the Executive Committee were submitted and accepted *en bloc* without discussion. In the next number of this journal (November 9) a report is published of the meeting of the Executive Committee, from which the names of those present are withheld. The business done appears to have been a series of resolutions condemning the Bill of what is described as a lay company, the College of Nursing, Limited—by Guarantee—if you please. We understand that publication of the names of the members present would destroy any weight which the resolutions in question might carry on their surface, and it seems to be a matter of some importance to the individuals who are members of the Society for the State Registration of Trained Nurses to realise the importance of first having before them the names of those present at any meeting of the Executive Committee, and to publish them for the information of members of Parliament and others who are interested in the State Registration of Nurses.

Meantime it is important that nurses generally should understand that the old Bill of the Fenwick party has been revived, that all attempts to secure unity have been wantonly frustrated by the supporters of that Bill, and that the College of Nursing has decided to proceed without delay to promote their own Bill, which provides for the inclusion on the first legal Register, without further fee, of all nurses on the College Register at the time of the passing of the Act, and, further, for the election of two-thirds of the General Nursing Council by the nurses upon the General Register under the Act. The Fenwick Bill contains no clause for the protection of trained nurses by providing that two-thirds of the General Nursing Council shall be elected by the nurses on the General Register. The number of nurses on the College Register is continuously growing, and should certainly amount to 15,000 at least by December 31 next.

The matron of a small fever sanatorium writes: During the year 1918 I limited my efforts at gardening to potatoes, artichokes, greens, onions, marrows, and runner beans. I also have a corner with parsley, mint, thyme, and sage. The ground was prepared, some of it, newly dug up, by the convalescent military patients, both officers and men. No manure was procurable, but an excellent crop resulted. The early potatoes were ready for consumption at the end of June, and the main crop in September. There was a yield of nearly a ton, enough to last the hospital four months. During the spring months there were greens for two months' consumption; the potato ground is now planted with cabbages and broccoli.

Our correspondent continues: Owing to the failure of the plum, damson, and greengage crop, much less jam was made, and no fruit was bottled. One cwt. of sugar was allotted me. The following quantities of preserves were made:—Rhubarb, 24 lb.; plum and apple, 56 lb.; marrow, 35 lb.; carrot marmalade, 14 lb.; and lemon marmalade, 30 lb. Fifty pounds of walnuts were pickled, 48 lb. of red cabbage, and 12 lb. of tomato chutney. Enough walnuts for Christmas use have been stored. Apples had to be used up quickly as there were few sound ones. This is a great loss; usually there are sufficient apples to tide over the winter. The carrot marmalade was made from carrots sent from a Harvest Thanksgiving, and the lemon marmalade was done when lemons were 2d. each. I reared twenty-six chickens (eleven were cockerels and were killed off for special diets) and nine ducklings. The ducklings are to be used for Christmas fare. My six hens laid a fair quantity

**ROUND THE HOSPITALS—(*continued*).**

of eggs, considering they were fed on scraps from the kitchens and wards, and that no grain was bought for them. The financial result of my year's poultry keeping I well send you at the end of the financial year.

We have not yet heard full particulars of the great Manchester bazaar in aid of the Nation's Tribute to Nurses. With characteristic generosity, Manchester and East Lancashire set itself the task of raising actually a tenth part of the £250,000 which it is estimated the endowment of the College of Nursing, with its Benevolent Fund, will require. And as we go to press the total reached is the splendid one of £6,629. All the hospitals in the area, under the inspiration of the Local Representative, Miss Sparshott, took part in making the bazaar a success, and there was never a doubt from the beginning as to how it would go. Victory was already in the air, and everyone was in high spirits, a condition the most favourable of all others to that lavish expenditure which stall-holders appreciate. A legitimate outlet for long-repressed impulses to spend was felt to be positively a thing to be grateful for. A feature of the bazaar was a letter from Florence Nightingale, dated December 26, 1887, addressed to Colonel Coates who was to have opened the bazaar on the first day but, being prevented, by way of compensation sent the letter to be put up to auction. It is in reply to an invitation to open a bazaar in aid of the Manchester Volunteer Medical Staff Corps. Miss Nightingale in it expressed her regret at her inability to be present, as she was a permanent invalid, and gave an interesting account of the early attempts of partially trained nurses in London to tend the sick poor before any practical system of district nursing was inaugurated.

Owing to the influenza epidemic the bazaar to be held this week by the Yorkshire Centre of the College at Leeds in aid of the Nation's Fund for Nurses has been postponed to December 12, 13 and 14!

The Bishop of London has been calling attention to the fact that the Salonika campaign, with its many episodes of heroism, has rather slipped out of the public eye. It will be a surprise to many of our readers to learn that no fewer than 1,600 nurses are at work there, with 30,000 patients to be attended to. During the long period of inaction fever patients were the most plentiful, but the hospitals are now full of the wounded sent down to the base over almost trackless mountain paths. Hospital construction has kept pace with the needs of the situation, and the doctors and nursing sisters are well supplied with the best appliances.

The managers of the Royal Infirmary, Edinburgh, have made a temporary grant in the form of a bonus for the next three years to their probationers. First-year probationers are to have a bonus of £7, making their salary £17. Second-, third-, and fourth-year probationers are to receive a bonus of £5, making salaries, £19, £23, and £30 respectively. The whole question of salaries and conditions of service is to be revised "at no very distant date." It is a big matter, considering the size and influence of the Royal Infirmary, which will naturally be expected to lead the way in promoting the welfare of nurses in the Scottish training-schools.

At the last session of the Cape Provincial Council an Ordinance was passed on nurses' salaries and pensions which is having a remarkable effect already in levelling up and standardising the payment of trained nurses in the Dominion. Members of institutions' staffs are graded according to rank and the size of the hospital, with the following results: Matrons' salaries, in addition to £12 in lieu of uniform, in fourth grade (up to twenty-five beds), £100-£140; third grade (up to 100 beds), £120-£170; second grade (up to 400 beds), £150-£220; first grade (over 400 beds), £250-£350; assistant matrons, Grade 2, £120-£170, and Grade 1, £150-£220; night superintendents, £5 less; sisters, £90 in first year, £100 in second year, thereafter by annual increments of £5 to £120; sisters in operating theatres and *x*-ray departments, £10 extra; staff nurse, £65, rising by annual increments of £5 to £90; probationers, £12, £24, £30. Compare this scale with that recently sanctioned by the Metropolitan Asylums Board.

It is often contended that prices rule so high at the Cape that it is impossible to compare salaries here and over there. It is to be noted, however, that these salaries are accompanied by free board, lodging, laundry, and uniform, so that the question of whether or no living costs more than in England does not enter. In fact, seeing that the allowance in lieu of board and lodging is fixed at £60 for a district nurse—precisely the same rate as that fixed by the Hertfordshire County Council, less their war bonus of £12—it may be inferred that the difference in cost of ordinary articles is not so great as is generally supposed. A valuable provision of the Ordinance relates to leave. It rules that a nurse is entitled to one month's leave every year, and further lays down that, after five years' service, and every five years after that, she may be given three months' leave on full pay and three months' on half-pay. This is a regulation which should bring new life into the Nursing Service.

The new schedule of salaries has been issued by the Metropolitan Asylums Board, and, as was anticipated, they show a very marked increase in the rate of remuneration for probationers as compared with the rest of the staff. Probationers are to begin at £22, increasing to £24 in fever (acute) and to £26 in children's hospitals; senior probationer, £31-37, and assistant nurse, Class I., the same. Staff nurses are to have £33, rising by £3 to £39; sisters £44, rising by £3 to £50; assistant matrons of hospitals and children's hospitals £80, rising by

**ROUND THE HOSPITALS—(*continued*).**

£5 to £95; second assistant matron, £70-£85; home sister, £55-£60; massage sister, £80-£100. It is very disappointing to find that the salaries of ward sisters are fixed at no higher rate than £44, a sum which compares very unfavourably with that now offered generally in first-class institutions.

A grievance acutely felt in some of the M.A.B. hospitals is swept away by the new schedule. On leave days nurses are to be allowed the value of the day's board on giving due notice. The cost of food on off-duty days has been felt as a recurring drain on the slender income, and the concession will be a great boon. The cost of board is reckoned at 2s. a day for male and female staff alike, a testimony to the improvement in housekeeping which has marked the M.A.B. institutions since war broke out.

The estimate made by the Hertfordshire County Council of a nurse's expenses for board and lodging is 20s. a week in a village, 23s. a week in towns. To this must be added the extra £1 a month given as war bonus to cover abnormal prices. That this estimate is not excessive is proved by the Government action in again raising the allowances to soldiers' wives, and by the Boards of Guardians (not lavish they), which pay up to 1s. a day at present for even tiny boarded-out children. The distinction between town and village is one of rent, for in towns the better shopping facilities make food cheaper. What excuse, then, have the Nursing Associations for keeping nurses at work on a salary of from £62 to £75, a rate which leaves nothing over when bare living expenses have been met? This, to our knowledge, is the sum given and uncomplainingly tolerated in certain districts. The County Nursing Associations should stop this abuse, and it is the duty of Queen Victoria's Jubilee Institute to rouse them to a sense of their shortcomings.

The West Suffolk Hospital, Bury St. Edmunds, is to be congratulated on a successful War Savings Association. Since it was inaugurated in February 1917 it has received considerably over £300 subscribed by the nursing and domestic staff for the purchase of War Savings Certificates. It is probable, notwithstanding sanguine forecasts, that the nation will have to go on saving for a long time after peace is declared, and the patriotic secretaries and treasurers of these Associations must continue to shoulder their burdens. Fresh Associations are being started every day, and it is certain no institution ought to stand out of this vital movement.

We learn with great satisfaction that the governors of the Ilford Emergency Hospital granted, at their last meeting, an increase of staff sufficient to allow the matron, Miss E. E. Green, to arrange for one day's rest in seven for all the nurses, supplementing it at the same time by a 25 per cent. increase on the salaries of the nursing staff. We believe that this concession of one day's rest in seven to the nursing staff has not hitherto been granted by the board of any small hospital, nor indeed so far has it come into action in any hospital down south to our knowledge. The Bristol Royal Infirmary led the way in England; the Aberdeen Royal Infirmary in Scotland. We wait with interest to see which hospital will first achieve this great reform in London.

The defective arrangements for enabling relatives to visit invalided members of the Nursing Services and officials of other women's War Services have long constituted a weak spot in an otherwise admirable organisation. Telegrams are now to be sent to the relatives in these cases, as to those of officers and soldiers. In future when the burial of an officer, nursing sister, or soldier is arranged by the military authorities a telegram will be sent, and the railway expenses of two persons will be provided free of cost, if desired, to the place of burial.

Miss Sara, who since war broke out has been Red Cross masseuse to the Herefordshire Branch of the B.R.C.S., has left for France to continue her labours there for the wounded. A gratuity of £50 has been granted her by the Finance Committee in recognition of her extraordinarily good work in the county where she has voluntarily undertaken thousands of cases. Herefordshire has done well in the war, and has recently voted £2,000 to the Central Funds, in addition to £1,300 ear-marked for their hut at Netley.

A nurse was arrested recently by a detective who had observed her, while selling flags for a war charity, pocketing part of the sums given to her. She has now been acquitted, the jury having stopped the case and found her "Not guilty" on hearing part of the evidence. She was able to show that she brought change with her and gave it from her pocket to purchasers of flags. And indeed it is difficult to see how flag-sellers can do otherwise. That she should have been exposed to the great annoyance of being watched and arrested is one of the strongest arguments we have had against Flag Days. With the end of the war let us hope that the street collection, so fruitful in stimulating dishonesty, may disappear from our midst. In spite of stringent regulations, children are still employed freely for this purpose, and in East Sussex pernicious begging-cards have been circulated broadcast to the elementary schools, with a request to the head teachers that they should be given to the children to tout for pennies. There are few worse ways of getting money than this.

☛ It is our invariable practice to pay for all items of news which we may publish. The minimum payment is 5s. Paragraphs of about twenty lines in length—that is to say, of 150 words or less—are preferred. Contributions should be addressed to The Editor, The Hospital, 28 and 29 Southampton Street, Strand, London, W.C. 2, and, to be in time for the current week's issue, should reach him by the first post on Mondays.

